# Anti-CD11b antibody treatment suppresses the osteoclast generation, inflammatory cell infiltration, and autoantibody production in arthritis-prone FcγRIIB-deficient mice

**DOI:** 10.1186/s13075-018-1523-1

**Published:** 2018-02-08

**Authors:** Mareki Ohtsuji, Qingshun Lin, Hideki Okazaki, Kazuko Takahashi, Hirofumi Amano, Hideo Yagita, Hiroyuki Nishimura, Sachiko Hirose

**Affiliations:** 1 Department of Biomedical Engineering, Toin University of Yokohama, 1614 Kurogane-cho, Aoba-ku, Yokohama, 225-8502 Japan; 2 Health and Life Science, Musashigaoka Junior College, Saitama, 355-0154 Japan; 3 Faculty of Health and Welfare, Kanagawa University of Human Services, Yokosuka, 238-8522 Japan; 4 Department of Rheumatology and Internal Medicine, Juntendo University School of Medicine, Tokyo, 113-8421 Japan; 5 Department of Immunology, Juntendo University School of Medicine, Tokyo, 113-8421 Japan

**Keywords:** Rheumatoid arthritis, FcγRIIB deficiency, Osteoclast, Monocytes, B cell activation

## Abstract

**Background:**

Previously we established an arthritis-prone FcγRIIB-deficient mouse strain (designated KO1). Anti-mouse CD11b mAb (5C6) has been reported to inhibit the recruitment of peripheral CD11b^+^ myelomonocytic cells from the blood to the inflammatory site. These cells include neutrophils and monocytes, both of which play important roles in the development of arthritis. Here we treated KO1 mice with 5C6 mAb in order to study its effect on arthritis development.

**Methods:**

To evaluate the disease-preventive effect of 5C6, 4-month-old preclinical KO1 mice were divided into three groups: the first treated with 5C6 for 6 months, the second treated with normal rat IgG for 6 months, as a control, and the third left untreated. Arthritis severity and immunological abnormalities were compared among the groups, along with transcriptional levels of several important arthritis-related factors in ankle joints, spleen, and peripheral blood cells.

**Results:**

The 5C6 treatment ameliorated arthritis in KO1 mice, showing decreases in inflammatory cell infiltration and osteoclast formation. Analysis of transcriptional levels in ankle joints revealed that compared with the two control groups, the 5C6-treated group showed downregulated expression of RANK, RANKL, MCP-1, RANTES, TNFα, and IL-6, and at the same time showed significantly up-regulated expression of the decoy receptor for RANKL, *i.e.* osteoprotegerin. In addition, the disease suppression was associated with the lower serum levels of autoantibodies, and the decreased frequencies of activated B cells and plasma cells. The expression levels of B cell activation/differentiation-related cytokines were suppressed in spleen and peripheral leukocytes of the 5C6-treated mice. Intriguingly, while untreated KO1 mice spontaneously developed marked monocytosis, the 5C6-treated mice showed the significantly down-regulated frequency of monocytes.

**Conclusions:**

The outcome of 5C6 treatment was complex, in which the 5C6-mediated disease-preventive effect is likely due on one hand to the decrease in the recruitment of inflammatory cells and osteoclast precursor monocytes from the periphery into the joints, and on the other hand to the suppression of B cell activation/maturation and of autoantibody production via the suppression of B cell stimulating cytokine production. The lower levels of these cytokines may be the secondary effect of the lower frequency of monocytes, since monocytes/macrophages are the major producers of these cytokines.

## Background

Rheumatoid arthritis (RA) is a chronic systemic autoimmune disease, characterized by synovitis with synovial lining cell hyperplasia and marked inflammatory cell infiltration in multiple joints in the initial stage; followed by marked pannus formation and progressive destruction of cartilage and bone mediated by augmented proliferation of activated osteoclasts. Osteoclasts are multinucleated giant cells positive for tartrate-resistant acid phosphatase (TRAP) and cathepsin K, and they resorb bone matrix [1, 2]. These cells differentiate from the osteoclast precursors of monocyte/macrophage lineage cells in the bone marrow [3] and in peripheral blood [4]. The process of osteoclastogenesis is controlled by the interaction of receptor activator of NF-κB (RANK) expressed on osteoclast precursors with its ligand RANKL expressed on synovial fibroblasts, osteoblasts, and T helper 17 (Th17) cells [5–7]. The RANKL-mediated osteoclastogenesis is counterbalanced by the physiologically expressed decoy receptor, osteoprotegerin (OPG) [8]. Accumulating evidence has indicated that high expression levels of proinflammatory cytokines, such as tumor necrosis factor α (TNFα), interleukin (IL)-1, IL-6, and IL-17, in inflamed synovial tissues may play pivotal roles in the processes of both synovitis and osteoclastogenesis [9].

We previously found that an inhibitory IgG Fc receptor IIB (FcγRIIB)-deficient mouse strain, originally established on 129 × C57BL/6 (B6) genetic background and backcrossed to B6 mice, designated KO1, spontaneously develops human RA-like disease features with marked synovitis and severe cartilage/bone destruction in multiple joints [10]. Signal blockade of IL-6 or TNFα markedly ameliorates arthritis in KO1 mice [11, 12]. Also, intriguingly, the severity of arthritis was positively associated with the increased frequency of peripheral monocytes in KO1 mice [11]. Since peripheral monocytes are composed of several subsets, including inflammatory cells and osteoclast precursors [4], the high frequency of peripheral monocytes may contribute to the up-regulation of both inflammatory cell infiltration and osteoclastogenesis in the arthritic joints of KO1 mice.

The rat anti-mouse CD11b mAb, 5C6, has been shown to inhibit recruitment of peripheral CD11b^+^ myelomonocytic cells into the inflamed lesion [13]. These CD11b^+^ myelomonocytic cells include neutrophils and monocytes, both of which play important roles in the initiation and progression of arthritis [11, 14]. We treated KO1 mice with 5C6 to evaluate the arthritis-preventive potential of 5C6 mAb in KO1 mice. The 5C6 treatment ameliorated arthritis in KO1 mice with decrease in inflammatory cell infiltrations, osteoclast formation, and RA-related cytokine production in the joint tissues. The 5C6 treatment also suppressed B cell activation/differentiation and autoantibody production. The possible mechanism in which 5C6 treatment ameliorates disease severity is discussed.

## Methods

### Mice

Arthritis-prone KO1 is a FcγRIIB-deficient B6 congenic line [10], obtained by backcrossing the originally constructed FcγRIIB-deficient mice on a hybrid (129 × B6) background into a B6 background for over 12 generations. C57BL/6 (B6) mice were purchased from Japan SLC, Inc., and used as the arthritis-free normal control. Only female mice were analyzed in the present study. All mice were housed under identical specific-pathogen-free conditions. All animal experiments were performed in accordance with the animal experiment guidelines of our university.

### Incidence and scoring of arthritis

Ankle joint swelling was examined by eye inspection twice a month after 5 months of age and arbitrarily scored as follows: 0, no swelling; 1, mild swelling; 2, moderate swelling; and 3, severe swelling. Scores for both ankle joints were summed for each mouse, and mice with a score of 2 or over were considered positive for arthritis. In addition to eye inspection, the degree of swelling in the region of the tarsal bones was measured at 10 months of age under anesthesia using a Vernier micrometer.

### *In vivo* administration of anti-CD11b mAb (5C6)

To examine the preventive effect of mAb 5C6 on the development of arthritis, 4-month-old preclinical KO1 mice were randomly divided into three groups. Each group of 15 mice was left untreated, treated with normal rat IgG (Sigma Chemical Co.), or treated with rat anti-mouse CD11b mAb (5C6, rat IgG2b [13]). Two hundred micrograms of rat IgG or 5C6 was administrated intraperitoneal (i.p.) twice a week for 6 months.

### Histopathology

Joint tissues were decalcified in 10% EDTA in 0.1 M Tris buffer (pH 7.4), fixed in 4% paraformaldehyde, and embedded in paraffin. Tissue sections were stained with hematoxylin/eosin, and also stained for TRAP using the TRAP/ALP stain kit (Wako Pure Chemical Industries Ltd.).

### Serum levels of antibodies

Serum levels of IgG anti-cyclic citrullinated peptide (CCP) antibodies were measured employing commercially available kits (Cosmic Corporation) using anti-mouse IgG second antibodies and were expressed as relative units according to the manufacturer’s instructions. Serum levels of rheumatoid factor (RF) were measured using an ELISA, as previously described [15]. Briefly, an ELISA plate pre-coated with mouse IgG Fc fragment (OEM Concepts) was incubated with appropriately diluted mouse serum samples, washed, and then incubated with peroxidase-conjugated rat anti-mouse κ chain antibodies (BD Biosciences Pharmingen). RF activity was expressed in units referring to a standard curve obtained by serial dilution of a standard serum pool from 4–6-month-old MRL/*lpr* mice containing 1000 unit activities/ml. Serum IgG anti-double-stranded (ds) DNA was measured using an ELISA plate pre-coated with 5 μg/ml calf thymus dsDNA (Sigma-Aldrich). DNA-binding activity was expressed in units as previously described [10].

### Flow cytometric analysis

Spleen cells were stained with phycoerythrin (PE)-labeled anti-B220 (RA3-6B2) mAb, allophycocyanin (APC)-labeled anti-CD69 (H1.2 F3), anti-CD138 (281-2), and anti-CD11c (HL3) mAbs, fluorescein isothiocyanate (FITC)-labeled anti-CD4 (RM4-5) and anti-CD11b (M1/70) mAbs, and FITC-labeled peanut agglutinin (PNA). For peripheral monocyte staining, peripheral leukocytes were stained with FITC-labeled anti-CD11b, PE-labeled anti-Gr-1 (RB6-8C5), and APC-labeled CD115 (AFS98) mAbs. Fluorescent-labeled reagents were purchased from Bay Bioscience (B220, CD4, CD11b, Gr-1, CD115), Bio Legend (CD69, CD138), BD Bioscience (CD11c), and Sigma-Aldrich (PNA). Stained cells were analyzed using a FACSAria cytometer and FlowJo software (Tree Star Inc.). Auto-fluorescence was considered as negative control. Compensation of spillover was performed according to the manufacturer’s instructiongei.

### Quantitative real-time PCR (qRT-PCR) analysis

Total RNA was isolated from ankle joint tissue of the tarsal bones containing soft tissue and bone/cartilage/marrow, from the spleen, and from peripheral leukocytes using QIAGEN RNeasy Lipid Tissue Minikit (Cat. number 74804). Briefly, ~ 25 mg of ankle joint tissue, spleen, or leukocyte pellet was added in 500 μl of QIAzol lysis reagent in a 2-ml tube containing 5-mm-diameter zirconia beads (Hirasawa YTZ-5) and homogenized on TissueLyser (Qiagen) for 1 min at 30 Hz. Total RNA was extracted from homogenized materials using Minikit according to the manufacturer’s instructions, and 0.5 μg of total RNA was used to synthesize the single-stranded cDNA using an oligo (dT)-primer with Superscript III First-Strand Synthesis kit (Invitrogen). The cDNA product was used for qRT-PCR. The data were normalized to β-actin as a reference. The primer pairs used and the length of PCR products are shown in Table 1. The quantity was normalized using 2^-∆∆CT^ methods.

**Table 1:** Primer pairs used in qRT-PCR and size of PCR products

Gene for	Forward	Reverse	Size
	(5’ to 3’)	(5’ to 3’)	(bp)
RANK	GCTGGCTACCACTGGAACTC	GTGCAGTTGGTCCAAGGTTT	182
RANKL	TGTACTTTCGAGCGCAGATG	AGGCTTGTTTCATCCTCCTG	160
OPG	TGAGTGTGAGGAAGGGCGTTA	CCATCTGGACATTTTTTGCAAA	130
MCP-1	AGGTCCCTGTCATGCTTCTG	TCTGGACCCATTCCTTCTTG	249
CX3CL1	CGCGTTCTTCCATTTGTGTA	CTGTGTCGTCTCCAGGACAA	169
RANTES	TCGTGCCCACGTCAAGGAGTATTT	TCTTCTCTGGGTTGGCACACACTT	107
TNFα	TATGGCCCAGACCCTCAC	GGTTGTCTTTGAGATCCATGC	110
IL-6	GAGGATACCACTCCCAACAGACC	AAGTGCATCATCGTTGTTCATACA	141
IL-17	TCTCTGATGCTGTTGCTGCT	GACCAGGATCTCTTGCTGGA	346
IL-10	CAGCCGGGAAGACAATAACT	GTTGTCCAGCTGGTCCTTTG	121
BAFF	TTGTCCAGCAGTTTCACAGC	CCGGTGTCAGGAGTTTGACT	160
IL-1β	GCCCATCCTCTGTGACTCAT	AGGCCACAGGTATTTTGTCG	230
BSF-3	CGAGCCTGACTTCAATCCTC	TACGTCGGAGTTCAGCTGTG	185
IFNγ	AAGACAATCAGGCCATCAGC	ATCAGCAGCGACTCCTTTTC	225
β-actin	TGGGTATGGAATCCTGTGG	GTACTTGCGCTCAGGAGGAG	200

*RANK* receptor activator of NF-κB, *RANKL* RANK ligand, *OPG* osteoprotogerin, *MCP-1* monocyte chemotactic protein-1, *RANTES* regulated on activation, normal T cell expressed and secreted, *BAFF* B-cell-activating factor of the tumor-necrosis-factor family, *BSF-3 B* cell stimulating factor, *IFN-*γ interferon-γ, *bp* base pairs

### Statistics

The Kaplan-Meier method was used to evaluate differences in the incidence of arthritis. Analysis of variance (ANOVA) was used to make comparisons among three or four groups of mice, and Student’s *t* test was used to compare two groups of mice. A value of *P* < 0.05 was considered as statistically significant.

## Results

### Suppressive effect of 5C6 treatment on severity of arthritis

**Fig. 1:**
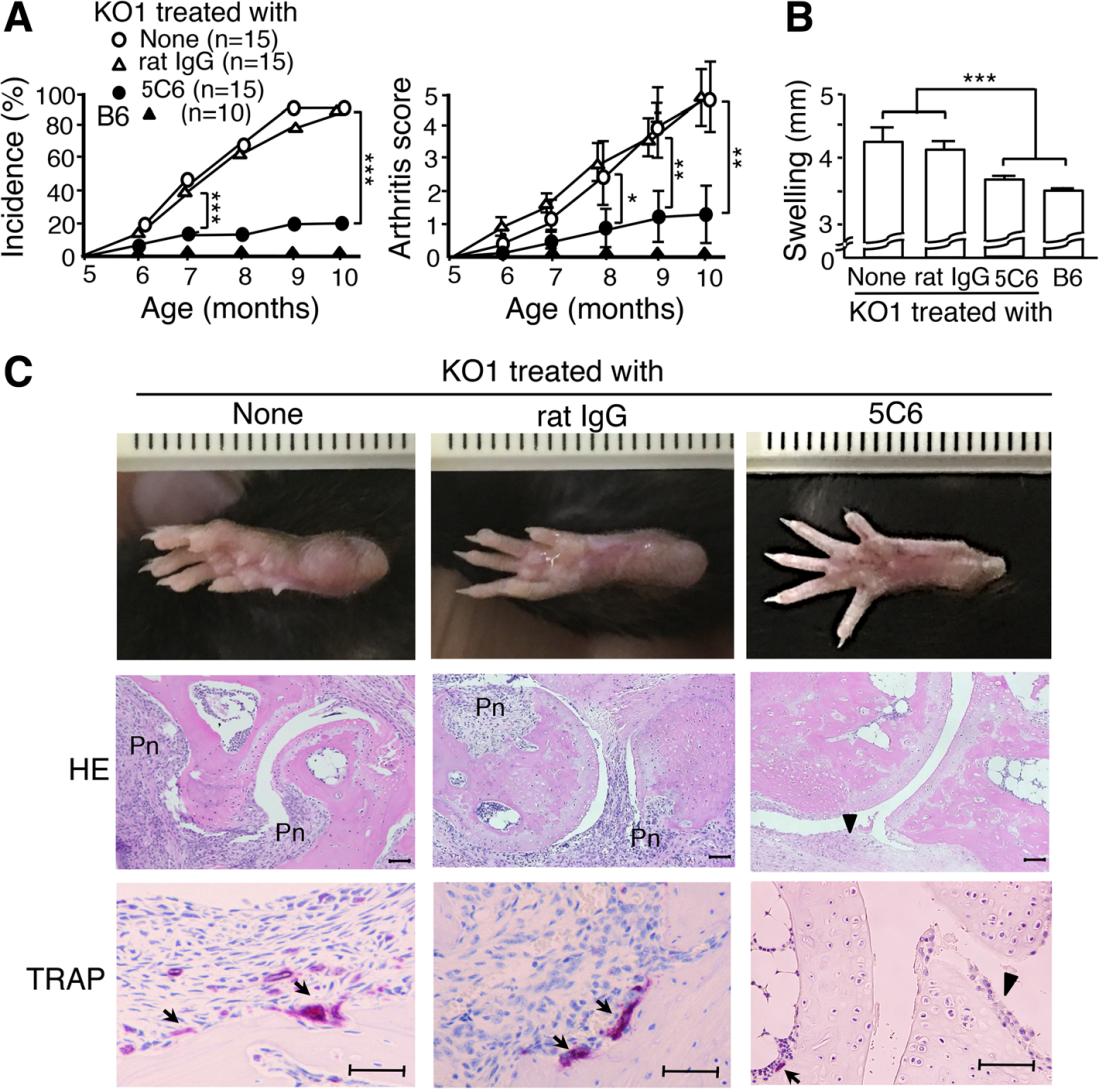
Preventive effect of anti-mouse CD11b monoclonal antibody (5C6) treatment. **a** Comparisons of the cumulative incidence of arthritis and the age-associated changes in arthritis score (mean and SE) among untreated (n = 15), normal rat IgG-treated (n = 15), 5C6-treated FcγRIIB-deficient mouse strain (KO1) (n = 15), and B6 mice (n = 10). The incidence was significantly lower in 5C6-treated mice, compared with those in untreated and normal rat IgG-treated mice after 7 months of age (Kaplan-Meier method ***P < 0.001), and the arthritis score was significantly suppressed in 5C6-treated mice, compared with those in untreated and normal rat IgG-treated mice after 8 months of age (analysis of variance (ANOVA) *P < 0.05, **P < 0.01). **b** Comparisons of the width of the ankle joints at the tarsal bones (mean and SE) of at least 6 mice at 10 months of age among untreated, normal rat IgG-treated, 5C6-treated KO1, and B6 mice. Statistical significance is shown (ANOVA ***P < 0.001). **c** Representative macroscopic and histopathological findings in the hind paws in untreated, normal rat IgG-treated, and 5C6-treated KO1 mice at 10 months of age. Untreated and normal rat IgG-treated KO1 mice had marked synovitis with inflammatory cell infiltration, pannus formation (Pn), tartrate-resistant acid phosphatase (TRAP)-positive osteoclast generation (arrowhead), and the destruction of cartilage and bone. The 5C6-treated KO1 mice only had mild synovial lining cell proliferation (triangle) and a few small TRAP-positive osteoclasts in bone marrow (arrowhead). Representative results obtained from six female mice in each group. Hematoxylin/eosin (HE) and TRAP staining. Bars = 50 μm

Four-month-old KO1 mice in the preclinical stage were divided into three groups, namely untreated, normal rat IgG-treated, and 5C6-treated groups. Figure 1a compares the cumulative incidence of arthritis and the age-associated changes in the arthritis score. Untreated KO1 mice spontaneously developed arthritis with bilateral swelling of the ankle joints after 5 months of age, and the severity of arthritis increased with age. KO1 mice treated with normal rat IgG developed severe arthritis similar to those observed in untreated KO1 mice. In contrast, in 5C6-treated KO1 mice the incidence and severity of arthritis were markedly suppressed from the initiation of arthritic inflammation. Although the incidence and severity were marginally higher in 5C6-treated KO1 mice compared with those in normal B6 mice, no significant difference was observed. Figure 1b shows the measurement of ankle joint width in the region of the tarsal bones at 10 months of age. The increased width due to joint inflammation observed in untreated and normal rat IgG-treated mice was significantly suppressed in 5C6-treated KO1 mice. Figure 1c shows representative macroscopic and histological findings in the hind paws in the three groups of mice at 10 months of age. Untreated and normal rat IgG-treated mice had marked swelling of the ankle joints, with severe synovitis and remarkable pannus formation, along with increases in the number of TRAP-positive osteoclasts at the resorption lacuna on the surface of cartilage and bone. The 5C6-treated KO1 mice developed no such distinctive changes and only had mild synovial lining cell proliferation. A few TRAP-positive small osteoclasts were observed in the bone marrow, but destructive changes of bone/cartilage were seldom observed.

### Effects of 5C6 treatment on serum autoantibody levels, lymphocyte activation status, and peripheral leukocyte frequencies

**Fig. 2:**
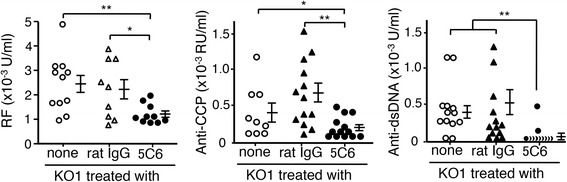
Comparisons of serum levels of rheumatoid factor (RF), and IgG autoantibodies against citric citrullinated peptide (CCP) and dsDNA among untreated, normal rat IgG-treated, and anti-mouse CD11b monoclonal antibody (5C6)-treated FcγRIIB-deficient mouse strain (KO1) mice at 8 months of age. The mean and SE are shown with statistical significance (analysis of variance *P < 0.05, **P < 0.01)

Figure 2 compares the serum levels of RF, and IgG autoantibodies against CCP and dsDNA among three groups of mice at 8 months of age. Levels of all these antibodies were significantly suppressed in 5C6-treated KO1 mice, compared with those in untreated and normal rat IgG-treated KO1 mice.

**Fig. 3:**
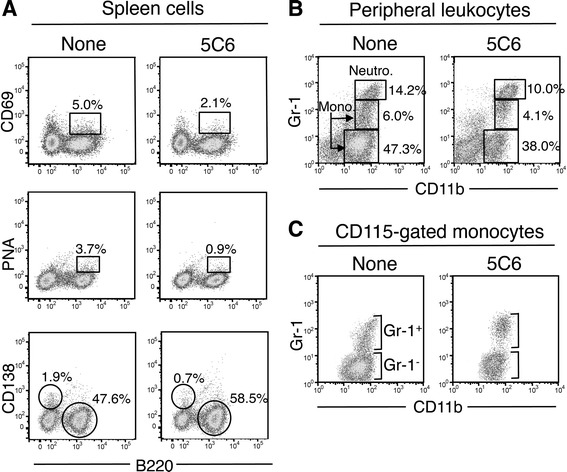
Representative flow cytometric profiles of spleen cells (**a**), peripheral leukocytes (**b**), and peripheral CD115^+^ monocytes (**c**) in untreated and anti-mouse CD11b monoclonal antibody (5C6)-treated FcγRIIB-deficient mouse strain (KO1) mice at 10 months of age. **a** Spleen cells were stained with fluorescent-labeled CD69/B220, peanut agglutinin (PNA)/B220, and CD138/B220. The frequencies of CD69^+^ activated B cells and of PNA^+^ germinal center B cells per total B cells are shown as squares. The frequencies of CD138^+^ plasma cells and B220^+^ B cells per total cells are shown as circles. **b** Peripheral leukocytes were stained with fluorescent-labeled Gr-1/CD11b. The frequencies of neutrophils, monocytes of Gr-1^+^ and Gr-1^-^ subsets per total cell are shown as squares. **c** Peripheral leukocytes were stained with fluorescent-labeled CD115/Gr-1/CD11b. Gr-1/CD11b expression profiles of CD115^+^ gated monocytes clearly show Gr-1^+^ and Gr-1^-^ monocyte subsets

We then examined the spleen weight, and the frequencies of spleen cell subpopulations and of peripheral leukocyte subsets in 10-month-old mice. As shown in Table 2, no differences were observed in the spleen weight among the three groups. Interestingly, in 5C6-treated mice, while the total B cell frequency was significantly higher, the frequencies of CD69^+^ activated B cells, PNA^+^ germinal center B cells, and CD138^+^ plasma cells were significantly lower, compared with those in the two control groups of mice. Figure 3a shows the representative flow cytometric profiles of spleen cells stained with CD69/B220, PNA/B220, and CD138/B220 in untreated and 5C6-treated KO1 mice. It is likely that in the control KO1 mice the accelerated B cell differentiation leads to the decrease in the frequencies of total B cells, and that this phenomenon is suppressed by 5C6 treatment. As for T cells, no significant differences were seen in the frequencies of total T cells or CD69^+^ activated T cells among the three groups of mice. The frequencies of CD11b^+^ macrophages and CD11c^+^ dendritic cells were comparable between untreated and 5C6-treated groups of mice. We also examined the effect of 5C6 treatment on the frequencies of peripheral leukocyte subsets. As 5C6 mAb has been shown to inhibit the recruitment of peripheral CD11b^+^ myelomonocytic cells into an inflamed lesion [13], it was suggested that the frequencies of peripheral CD11b^+^ cells including neutrophils and monocytes might be increased in 5C6-treated KO1 mice. However, our present study showed that the frequency of neutrophils tended to be decreased in 5C6-treated KO1 mice, although there was no statistical significance. Moreover, the frequency of monocytes, especially Gr-1^-^ monocytes, was significantly decreased in 5C6-treated KO1 mice (Table 2). Thus, in addition to the inhibition of CD11b^+^ myelomonocytic cell recruitment from vessels, 5C6 may have been somewhat cytotoxic in our experiment. The original report showed that 5C6 did not induce cell death [13]. This discrepancy may be due to our injection protocol, twice a week for 6 months, which is different to the single injection used in the original study. The representative flow cytometric profiles of peripheral leukocytes stained with Gr-1/CD11b are shown in Fig. 3b. Neutrophils had the highest Gr-1 expression level, while monocytes were divided into two populations, Gr-1^+^ and Gr-1^-^ subsets. These two monocyte subsets were clearly shown by staining peripheral leukocytes with CD115/Gr-1/CD11b and by examining Gr-1/CD11b expression profiles of CD115^+^ monocytes (Fig. 3c).

**Table 2:** Spleen weight and frequencies of spleen cell subpopulations and peripheral leukocyte subsets in untreated, normal rat IgG-treated, and 5C6-treated KO1 mice^a^

	Treatment		
	None	Rat IgG	5C6
Spleen weight (g)	0.20 ± 0.05	0.19 ± 0.04	0.19 ± 0.02
Spleen cell populations (%)			
B220^+^B cells/total cells	48.0 ± 2.7	47.7 ± 3.3	56.4 ± 1.9^b^
CD69^+^B220^+^ B cells/total B cells	5.3 ± 1.9	4.5 ± 1.0	2.7 ± 0.2^b^
PNA^+^B220^+^ B cells/total B cells	3.8 ± 1.2	3.9 ± 0.5	1.2 ± 0.1^c^
CD138^+^plasma cells/total cells	2.1 ± 0.5	1.7 ± 0.2	1.1 ± 0.2^d^
CD4^+^T cells/total cells	17.5 ± 0.4	16.2 ± 0.8	17.3 ± 0.7
CD69^+^CD4^+^ T cells/total T cells	35.9 ± 4.2	29.9 ± 2.5	34.64 ± 1.53
CD11b^+^ cells/total cells	12.0 ± 2.5	ND	9.1 ± 1.5
CD11c^+^ cells/total cells	6.9 ± 1.3	ND	5.8 ± 0.6
Peripheral blood (%)			
Neutrophils/total cells	16.8 ± 1.7	ND	9.5 ± 2.4
Monocytes/total cells	54.0 ± 2.3	50.5 ± 11.9	41.3 ± 1.8^b^
Gr-1^+^ monocytes/total cells	6.2 ± 1.4	ND	4.4 ± 0.6
Gr-1^-^ monocytes/total cells	47.8 ± 1.7	ND	37.0 ± 1.8^e^

*ND* not defined, *5C6* anti-mouse CD11b monoclonal antibody, *KO1* FcγRIIB-deficient mouse strain

^a^Values are the mean ± SEM of at least 6 female mice aged 10 months

^b^Differences were statistically significant versus untreated KO1 and versus normal rat IgG-treated KO1 (analysis of variance (ANOVA) *P* < 0.05)

^c^Differences were statistically significant versus untreated KO1 and versus normal rat IgG-treated KO1 (ANOVA *P* < 0.01)

^d^Differences were statistically significant versus untreated KO1 (ANOVA *P* < 0.01) and versus normal rat IgG-treated KO1 (ANOVA *P* < 0.05)

^e^Difference was statistically significant versus untreated KO1 (Student’s *t* test *P* < 0.05)

### qRT-PCR analysis for mRNA expression levels of RANK/RANKL/OPG and arthritis-related cytokines in ankle joints

**Fig. 4:**
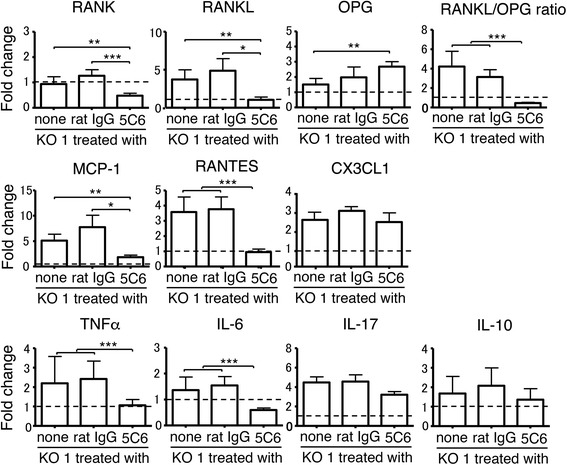
Comparisons of mRNA expression levels of receptor activator of NF-κB (RANK), RANK ligand (RANKL), osteoprotogerin (OPG), RANKL/OPG ratio, monocyte chemotactic protein-1 (MCP-1), regulated on activation, normal T cell expressed and secreted (RANTES), CX3CL1, TNFα, IL-6, IL-17, and IL-10 in the ankle joints analyzed by qRT-PCR among untreated, normal rat IgG-treated, and monoclonal antibody (5C6)-treated FcγRIIB-deficient mouse strain (KO1) mice at 10 months of age. Values in each of the three groups of mice were evaluated as fold change compared with the expression levels in B6 mice (broken line). Data are shown as mean + SEM of six mice from each group. Statistical significance is shown (analysis of variance *P < 0.05, **P < 0.01, *** *P* < 0.001)

To understand the mechanism of the suppression of arthritis in 5C6-treated KO1 mice, transcription levels of RANK, RANKL, OPG, and several important arthritis-related cytokines in ankle joint tissues were compared among the three groups of mice by qRT-PCR analysis using mRNA extracted from tarsal bones of the mice at 10 months old. Data on normal B6 mice were used as a relative control, and the expression levels in the three groups of mice were evaluated as fold change compared with the level in B6 mice tentatively designated as 1. As shown in Fig. 4, compared with untreated and normal rat IgG-treated KO1 mice, the expression levels of RANK and RANKL were significantly lower in 5C6-treated KO1 mice. In contrast, the OPG expression level in 5C6-treated KO1 mice was significantly higher than that in untreated KO1 mice. Furthermore, when calculating the RANKL/OPG ratio, the average ratio in 5C6-treated KO1 mice was much lower compared with those in the two control groups of mice.

Three chemokines, MCP-1, regulated on activation, normal T cell expressed and secreted (RANTES), and CX_3_C chemokine ligand 1 (CX3CL1), have been shown to play important roles in the pathogenesis of RA [16]; therefore we compared their expression levels in ankle joint tissue. The expression MCP-1 and RANTES, but not CX3CL1, was down-regulated in 5C6-treated mice, compared with those in the two control groups of mice (Fig. 4). Thus, it appears that MCP-1 and RANTES play pivotal roles in the inflammatory process in the arthritic joints of KO1 mice.

Figure 4 also compares the expression levels of TNFα, IL-6, IL-17, and IL-10 among the three groups of mice. The levels of TNFα and IL-6 were markedly down-regulated in 5C6-treated mice, compared with the levels in untreated and normal rat IgG-treated KO1 mice. No significant differences were observed in IL-17 and IL-10 expression levels among the three groups of mice. These findings are consistent with our previous reports showing that both IL-6 and TNFα are indispensable in the pathogenesis of arthritis in KO1 mice [11, 12].

### Effect of 5C6 treatment on B cell activation

**Fig. 5:**
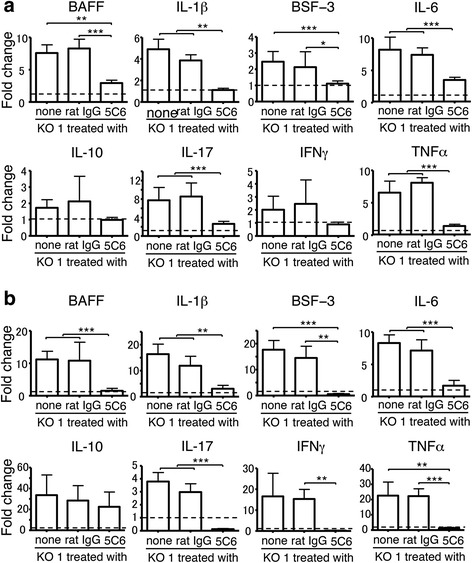
Comparisons of mRNA expression levels of B-cell-activating factor of the tumor-necrosis-factor family (BAFF), IL-1β, B cell stimulating factor-3 (BSF-3), IL-6, IL-10, IL-17, interferon-γ (IFN-γ), and TNFα in spleen (**a**) and peripheral leukocytes (**b**) analyzed by qRT-PCR among untreated, normal rat IgG-treated, and monoclonal antibody (5C6)-treated FcγRIIB-deficient mouse strain (KO1) mice at 10 months of age. Values of each of the three groups of mice were evaluated as fold change compared with the expression levels in B6 mice (broken line). Data are shown as mean + SEM of six mice from each group. Statistical significance is shown (analysis of variance *P < 0.05, **P < 0.01, *** *P* < 0.001)

As shown in Table 2, B cells were activated and differentiated in untreated and rat IgG-treated KO1 mice, and these were significantly suppressed in 5C6-treated KO1 mice. To evaluate the effect of 5C6 treatment on the expression levels of B cell activation/differentiation-related cytokines, such as B-cell-activating factor of the tumor-necrosis-factor family (BAFF), IL-1β, B cell stimulating factor-3 (BSF-3), IL-6, and IL-10, qRT-PCR analysis was performed using mRNA extracted from the spleens of the 10-month-old mice. Data on normal B6 mice were used as a relative control. As shown in Fig. 5a, the expression levels of the former four cytokines were markedly up-regulated in untreated and rat IgG-treated mice, while the levels were significantly suppressed by 5C6 treatment. We also compared the expression levels of IL-17, IFN-γ, and TNFα among the three groups of mice. The levels of IL-17 and TNFα were up-regulated in the control groups, and were significantly suppressed in 5C6-treated KO1 mice.

It has been shown that in lupus-prone mice the frequency of peripheral monocytes correlates well with the serum level of autoantibodies [17]. Although the reason for this correlation remains to be clarified, it is feasible to speculate that peripheral monocytes may function to induce B cell activation. To examine this possibility, B cell activation/differentiation-related cytokines were analyzed by qRT-PCR using mRNA extracted from peripheral leukocytes. As shown in Fig. 5b, the expression of BAFF, IL-1β, BSF-3, and IL-6 was up-regulated in the two control groups of KO1 mice, and significantly suppressed in 5C6-treated KO1 mice. The expression of IL-17, IFN-γ, and TNFα was also up-regulated in the control groups of mice, and suppressed in 5C6-treated mice. The tendency found in peripheral leukocytes was almost the same as that observed in the spleen. Taken together, it is likely that the decrease in the expression of B cell activation/differentiation-related cytokines, IL-17, and TNFα in the spleen in 5C6-treated KO1 mice is due to the decreased frequency of peripheral monocytes due to 5C6 treatment as shown in Table 2, and also due to the inhibited migration of peripheral monocytes into the spleen.

## Discussion

In arthritis-prone KO1 mice, the 5C6 treatment initiated at the preclinical stage markedly suppressed the incidence and severity of arthritis from the early stage of the disease. This mAb has been shown to inhibit recruitment of peripheral CD11b^+^ myelomonocytic cells into the inflammatory site with no cytotoxic effect [13]; however, in our experiment, the repetitive 5C6 treatment had a somewhat cytotoxic effect and induced decreased frequencies of myelomonocytic cells in the periphery. The 5C6-mediated disease suppression in the initiation stage of arthritis seems to be due to the decreased CD11b^+^ inflammatory cell migration into the joint tissues, since 5C6-treated mice had mild synovial lining cell proliferation but not inflammatory cell infiltration. In the later stage, KO1 mice developed the marked cartilage and bone destruction associated with severe synovitis, pannus formation, and up-regulated TRAP-positive osteoclast generation at the bone resorption lacuna. These destructive features were seldom observed in 5C6-treated KO1 mice due to the lack of osteoclast precursor monocyte recruitment into the joint tissues. The disease suppression was associated with down-regulation of serum RF and of IgG autoantibodies against CCP and dsDNA. These findings clearly demonstrate that, in addition to the effect on the decreased migrations and frequencies of peripheral CD11b^+^ myelomonocytic cells including inflammatory cells and osteoclast precursors, 5C6 mAb also exerts an inhibitory effect on B cell activation/differentiation.

RANK and RANKL expression was suppressed in the joint tissue in 5C6-treated KO1 mice, while OPG was up-regulated. The decrease in RANK expression is likely due to the decreased number of RANK-expressing osteoclast precursors. The decreased RANKL expression in 5C6-treated KO1 mice is thought to be due to decreased IL-6 expression, as IL-6 signals have been shown to directly induce RANKL expression in fibroblast-like synoviocytes from patients with RA [18]. As OPG is a decoy receptor for RANKL and suppresses RANKL-mediated osteoclastogenesis [8], the increased OPG expression and the lower RANKL/OPG ratio are consistent with the suppression of osteoclastogenesis in the joint tissues of 5C6-treated KO1 mice. OPG is produced by a variety of cells including osteoblasts, B cells, dendritic cells, and vascular endothelial cells [19, 20], and the marked decrease in OPG expression in arthritic joints has been suggested as a result of the exhaustion of OPG production due to chronic stimulation by TNFα over the extended period [20]. Consistently, synovial OPG expression has been shown to be increased in patients with RA treated with anti-TNFα [21] and in TNFα-deficient KO1 mice [11]. Thus, the increased level of OPG in 5C6-treated mice may be due to lower expression of TNFα.

RA is an autoantibody-mediated autoimmune disease, in which IgG immune complexes (ICs) trigger inflammatory myelomonocytic cell activation via IC-binding to the FcR γ chains of stimulating IgG Fc receptors [22, 23]. These activated inflammatory cells produce several inflammatory cytokines including chemokines, which induce additional inflammatory cell infiltration in the joint tissues. Our arthritis-prone KO1 mice lack the expression of inhibitory FcγRIIB molecules [10], which are usually expressed on B cells and myelomonocytic cells. The FcγRIIB molecules inhibit the activation signals mediated by the B cell antigen receptors on B cells and by the FcR γ chains on myelomonocytic cells [22, 23]. The lack of FcγRIIB expression on B cells induces the breakdown of self-tolerance and the production of IgG autoantibodies [24]. The resultant IgG ICs formed in the joint tissues stimulate myelomonocytic cells via the FcR γ chains, and this process is augmented in KO1 mice. In 5C6-treated KO1 mice, the expression levels of chemokines such as MCP-1 and RANTES were significantly down-regulated, indicating that these cytokines play important roles in the pathogenesis of arthritis in KO1 mice. Intriguingly, it has been shown that once RANK^+^ osteoclast precursor monocytes are activated via membrane-bound RANKL, it leads to increased expression of MCP-1 and RANTES, and these chemokines act as chemical signals, attracting other monocytes to the site of RANKL expression. These chemokines promote fusion of monocytes with RANKL-stimulated osteoclast precursors, resulting in the generation of multinucleated mature osteoclasts [25].

Peripheral monocytes are composed of heterogeneous populations and could mainly be subdivided into two phenotypically and functionally distinct subsets [4, 26]. In mice, monocytes are clearly divided into two populations, namely Gr-1^+^ and Gr-1^-^ subsets. The former is CX3CR1^low^CCR2^+^ and is called an “inflammatory” subset that is actively recruited to inflamed tissues via MCP-1, the ligand for CCR2, whereas the latter is CXCR3^high^CCR2^-^ and called a “resident” subset that is recruited to non-inflamed tissues. Intriguingly, in lupus-prone mice the Gr-1^-^, but not the Gr-1^+^ subset is selectively expanded and expresses the stimulatory FcγRIV, suggesting that the Gr-1^-^ subset may be in a more active stage than the Gr-1^+^ subset, and may contribute to kidney damage [27]. Compared with normal B6 mice with monocyte frequencies below 10% [17], our arthritis-prone KO1 mice develop marked monocytosis with frequencies above 50%, mainly composed of the Gr-1^-^ subset. The 5C6 treatment especially suppressed the frequency of Gr-1^-^ monocytes, suggesting the important role of the Gr-1^-^ subset in osteoclastogenesis. Thus, the originally classified Gr-1^-^ “resident” subset may contain functionally different cell types, one responsible for lupus nephritis and another for osteoclastogenesis in the joints. However, there are conflicting results reported on the cell types that can give rise to osteoclasts. Yao et al. [28] report that the Gr-1^-^ subset is the major precursor of osteoclasts. In contrast, Seeling et al. [29] showed that the Gr-1^+^ inflammatory monocyte subset represents the major precursor of osteoclasts, and that these Gr-1^+^ monocytes differentiated into mature osteoclasts paralleled by up-regulation of FcγRIV expression upon *in vitro* activation via RANKL. They also showed that the cross-linking of activating FcγRIV by ICs was critical for osteoclast development. Given the important role of FcγRIV in IC-mediated stimulation, Gr-1^-^FcγRIV^+^ monocytes may contribute to the initial step of inflammatory responses, including MCP-1 expression, in the arthritic lesion, and then Gr-1^+^CCR2^+^ monocytes are attracted by MCP-1, resulting in the fusion with activated Gr-1^-^FcγRIV^+^ monocytes to generate multinucleated mature osteoclasts, as suggested [25].

Genetic studies using lupus-prone mice showed that the frequency of peripheral monocytes correlates well with the serum level of autoantibodies [17]. In the present study, 5C6 treatment down-regulated the frequency of the Gr-1^-^ monocyte subset, along with decreased expression of BAFF, IL-1β, BSF-3, and IL-6 in the periphery, suggesting that Gr-1^-^ monocytes are the major producer of these cytokines in KO1 mice. BAFF enhances B cell survival and immune responses [30]. Although the important roles of IL-1β in innate immune responses and T cell differentiation are known [31], IL-1β is also well-recognized as an essential cytokine for antibody production and B cell proliferation [32, 33]. BSF-3, also reported as cardiotrophin-like cytokine, belongs to the IL-6 family and strongly stimulates antibody production by B cells [34, 35]. These B cell activation/differentiation-related cytokines may play a pivotal role in B cell activation and consequently in the pathogenic autoantibody production in KO1 mice. Taken together, the blocking of monocyte function may be an additional possible therapeutic strategy for rheumatoid arthritis.

## Conclusions

Our originally established FcγRIIB-deficient mouse strain (designated KO1) develops severe arthritis resembling human RA. Taking advantage of treatment of KO1 mice with the mAb 5C6, we showed that 5C6 treatment results in not only amelioration of arthritis but also decrease in autoantibody production. The amelioration of arthritis was thought to be due to the decreased number and recruitment into joint tissues of peripheral monocytes, including inflammatory cells and osteoclast precursors. The down-regulated autoantibody production was thought to be due to the decreased frequencies of monocytes, which have the potential to produce B cell activating cytokines. The present findings indicate that the blocking of monocyte function could be an additional possible therapeutic strategy for rheumatoid arthritis.

## Abbreviations

5C6: Anti-mouse CD11b monoclonal antibody
ANOVA: Analysis of variance
B6: C57BL/6
BAFF: B-cell-activating factor of the tumor-necrosis-factor family
BSF-3: B cell stimulating factor-3
CCP: Cyclic citrullinated peptide
CX3CL1: CX_3_C chemokine ligand 1
FcγRIIB: IgG Fc receptor IIB
ELISA: Enzyme-linked immunosorbent assay
ICs: Immune complexes
IFN-γ: Interferon-γ
IL: Interleukin
KO1: FcγRIIB-deficient mouse strain
mAb: monoclonal antibody
MCP-1: Monocyte chemotactic protein-1
OPG: Osteoprotegerin
PNA: Peanut agglutinin
RA: Rheumatoid arthritis
RANK: Receptor activator of NF-κB
RANKL: RANK ligand
RANTES: Regulated on activation, normal T cell expressed and secreted
RF: Rheumatoid factor
Th17: T helper 17
TNFα: Tumor necrosis factor α
TRAP: Tartrate-resistant acid phosphatase
